# A Review on Experimental Measurements for Understanding Efficiency Droop in InGaN-Based Light-Emitting Diodes

**DOI:** 10.3390/ma10111233

**Published:** 2017-10-26

**Authors:** Lai Wang, Jie Jin, Chenziyi Mi, Zhibiao Hao, Yi Luo, Changzheng Sun, Yanjun Han, Bing Xiong, Jian Wang, Hongtao Li

**Affiliations:** Department of Electronic Engineering, Tsinghua University, Beijing 100084, China; Jin-J15@mails.tsinghua.edu.cn (J.J.); mczy15@mails.tsinghua.edu.cn (C.M); zbhao@tsinghua.edu.cn (Z.H.); luoy@tsinghua.edu.cn (Y.L.); czsun@tsinghua.edu.cn (C.S.); yjhan@tsinghua.edu.cn (Y.H.); bxiong@tsinghua.edu.cn (B.X.); wangjian@tsinghua.edu.cn (J.W.); lihongtao@tsinghua.edu.cn (H.L.)

**Keywords:** light-emitting diodes (LEDs), efficiency droop, GaN, InGaN, multiple quantum wells (MQWs), carrier lifetime

## Abstract

Efficiency droop in GaN-based light emitting diodes (LEDs) under high injection current density perplexes the development of high-power solid-state lighting. Although the relevant study has lasted for about 10 years, its mechanism is still not thoroughly clear, and consequently its solution is also unsatisfactory up to now. Some emerging applications, e.g., high-speed visible light communication, requiring LED working under extremely high current density, makes the influence of efficiency droop become more serious. This paper reviews the experimental measurements on LED to explain the origins of droop in recent years, especially some new results reported after 2013. Particularly, the carrier lifetime of LED is analyzed intensively and its effects on LED droop behaviors are uncovered. Finally, possible solutions to overcome LED droop are discussed.

## 1. Introduction

GaN-based light-emitting diodes (LEDs) have achieved remarkable developments in the last two decades. They have already changed the human’s daily life significantly for their wide applications in liquid-crystal display (LCD) back-lighting, large screen display and general lighting [1,2,3]. That is why the Nobel Prize in Physics in 2014 was awarded to three scientists who did pioneering work on GaN-based LEDs [4]. Although the present commercial LEDs have high enough efficiency, they are still suffering from the efficiency declining when injection current density increases, which is known as efficiency droop [5,6,7]. This issue perplexes the LED industry because it has to enlarge the chip size to reduce the current density in high-power lighting application, which increases the cost. Recently, some new applications make LED droop more outstanding, e.g., LED-based visible light communication (VLC), which can realize ubiquitous wideband wireless access [8]. In VLC, to achieve a high modulation frequency, the LED should be better to work in a small chip size and under a higher current density, at least 10 times higher than the normal work point, to diminish the capacitance and shorten the carrier lifetime [9].

Meanwhile, efficiency droop effect also attracts the interests from academia [10,11,12,13,14,15,16,17,18,19,20,21,22,23,24,25,26,27,28,29,30,31,32,33,34,35,36,37,38,39,40,41,42,43,44,45,46,47,48,49,50,51,52,53,54,55,56,57,58,59,60,61,62,63]. Unlike that in conventional III–V semiconductors, this phenomenon in GaN-based LED has little relation to heat, which can be certified by the fact that the droop behaviors seem almost the same under CW and pulsed injection [7,11]. The earliest publications concerning LED droop appeared ten years ago [10,11,12,13,14,15,16]. From then on, there are many reports focusing on this topic. It is worth mentioning that, in 2013, two review papers summarized the research progresses until that time [6,7], including the possible mechanisms of LED droop and countermeasures. The researchers have reached a consensus that the external quantum efficiency (EQE) droop is induced by internal quantum efficiency (IQE) rather than light extraction efficiency (LEE), wherein EQE = IQE × LEE. However, the IQE of a LED under electrical injection is determined by both carrier radiative recombination efficiency (RRE) and carrier injection efficiency (CIE). The former represents the proportion of carriers emitting light inside the active region while the latter denotes the proportion of carriers injected into the active region. Consequently, the bone of contention is whether the origin of droop is the internal loss inside active region through some non-radiative recombination processes, or the injection loss outside active region, especially carrier non-radiative recombination in the p-type region of LED due to the asymmetrical injection of electrons and holes. The main proposed mechanisms for internal loss are carrier delocalization [29,37,40,48,61] and Auger recombination [12,15,25,35,47,49,52,54,57], while those for injection loss includes polarization-enhanced electron leakage, electron overflow and poor hole injection [11,18,19,20,22,24,32,33,34,36,38,39,41,42,46,51,60,62,63]. In 2013, there was also a milestone paper on first direct observation of Auger electrons in LEDs [54], which seems to answer the debate on the origin of droop perfectly. From then on, the study boom began to fade away gradually. However, the explanation that Auger recombination is dominantly responsible for LED droop is still not accepted by all researchers in this field. Some subsequent investigations remained to query the conclusion [60,62,63].

From the above background, it can be seen that the study on efficiency droop strongly relies on experimental measurements of LED as well as the physical model behind the measurement results. In the present review, several representative experimental measurements for understanding efficiency droop are introduced, including the basic principle, experimental results and possible limitations. It expects to bring some enlightenment to the researchers in this field, through summarizing the recent progresses on droop study. Moreover, solutions to overcome droop effect are also discussed based on the analysis.

## 2. ABC Model

The experimental measurements cannot tell the origin of efficiency droop directly, so the physical model is required to build the link between experimental results and droop. The ABC model is the most popular one to analyze the experimental phenomena, which can generally express the IQE of LED ($\eta_{\mathrm{int}}$) as

(1) $$\eta_{\mathrm{int}}=\eta_{\mathrm{inj}}\eta_{\mathrm{rad}}=\eta_{\mathrm{inj}}\frac{Bn^{2}}{An+Bn^{2}+Cn^{3}},$$

where *η*_inj_ is CIE and *η*_rad_ is RRE. *A*, *B*, *C*, and *n* represent Schockley–Read–Hall (SRH) recombination, radiative recombination, Auger recombination coefficients and carrier concentration in active region, respectively. It is should be pointed out that, in some semiconductors [64,65], radiative recombination can be described as *Bn*, wherein *n* denotes the exciton concentration. However, according to the latest carrier dynamics study in InGaN multi-quantum-well (MQW) [66], the radiative recombination is bimolecular recombination rather than exciton recombination and should be described as *Bn*^2^. The work by Badcock et al. has pointed out that it is the uncorrelated localization nature of electrons and holes that leads to a *Bn*^2^ recombination description [67]. From the work by Schulz et al., it can be seen that the holes and electrons are localized separately, not only in the growth direction but also in the QW plane [68], which will reduce the degree of correlation between electrons and holes. Therefore, *Bn*^2^ are widely-accepted by almost all of the important publications analyzing the IQE or efficiency droop in InGaN-based LEDs.

From Equation (1), it is easy to understand why it is challengeable to study the droop effect. First, there is not a widely-accepted method to obtain the accurate $\eta_{\mathrm{int}}$ under different current densities (*J*). One has to calculate it through the measured EQE and an estimated LEE value according to simulation or experience. Second, it is hard to distinguish the contribution of CIE and RRE to IQE, which is just the debate on LED droop mentioned in Section 1. People supporting internal loss believe that $\eta_{\mathrm{inj}}$ is almost constant (usually nearly 100%), and then the droop can only be induced by *η*_rad_. Auger recombination (*Cn*^3^) dominates if *n* is high or coefficient *C* is large, while carrier delocalization dominates if coefficient *A* increases and coefficient *B* decreases with the increase of current density. On the other hand, the people supporting injection loss believe that $\eta_{\mathrm{inj}}$ should decrease with current density. In addition, the carrier concentration *n* is determined by the current density *J*, thickness of active region *d* and carrier lifetime *τ* as

(2) $$n=\frac{\eta_{\mathrm{inj}}J\tau }{qd}\;.$$

Equation (2) builds a link between carrier concentration and current density. Usually, only the latter can be obtained directly in experiment. To obtain the former for droop analysis, both carrier lifetime and thickness of active region are required.

## 3. Electroluminescence (EL)

When we talk about efficiency droop, it means the EL droop. Thus, using Equation (1) to fit the experimental efficiency-current curve is the most direct way to understand droop. However, the actual carrier concentration is unknown only according to current density. For simplification, Galler et al. assumed $\eta_{\mathrm{inj}}$ is 100% and all the carriers are injected into the active region [69]. Then, carrier concentration can be reflected by current density. Consequently, Auger recombination is responsible for droop and coefficient *C* can be deduced, which is a deserved result that when injection loss is ignored. Liu et al. found *C* = 5.7 × 10^−29^ cm^6^/s in LED grown on sapphire substrate while *C* = 3 × 10^−30^ cm^6^/s in the one grown on free-standing GaN substrate [70], by using the same assumption and fitting. Obviously, this assumption is arbitrary. Dai et al. believed that Auger recombination is impossibly the unique reason responsible for droop by analyzing the shape of efficiency-current curve [34]. However, if injection loss is taken into account in fitting, it is hard to find a widely-accepted analytical expression of $\eta_{\mathrm{inj}}$. In some publication [71], the injection loss is also considered dependent on current density *J*, and expressed as *kJ^b^*, wherein *k* and *b* are fitting parameters. This assumption is easy to handle in fitting, but its validity in physics is still questionable.

### 3.1. Temperature Dependent Electroluminescence (TDEL)

TDEL is a direct and simple way to observe the droop behaviors. Almost all the reported TDEL show that efficiency droop becomes severer under low temperature [32,37,39,72]. This phenomenon is usually regarded as a strong evidence to verify that carrier injection loss is responsible for droop. Because the carrier injection asymmetry is exacerbated under low temperature due to the poor hole ionization, whereas the Auger recombination coefficient should decrease with the temperature [37]. Under low temperature, the efficiency reaches it maximum at a smaller current density, implying the onset of droop has been advanced. However, it should be noticed that a same current density may correspond to different carrier concentrations at different temperatures, because carrier lifetime will become longer when temperature decreases. Thus, TDEL behaviors may not be simply used to verify the origin of droop.

Piprek analyzed an LED by using numerical simulation [73]. The main evidence he excluded carrier leakage as primary cause of droop is the temperature-dependent droop behaviors. As mentioned above, this phenomenon is easy to understand through temperature-dependent carrier lifetime and non-radiative SHR recombination. Under high temperature, the non-radiative recombination becomes more serious, so the carrier lifetime becomes shorter and also the RRE becomes lower. As carrier lifetime becomes shorter, the carrier concentration in QW will be smaller than that under low temperature even if the current density is the same. Thus, temperature-dependent droop behaviors are similar to those in LEDs with different dislocation densities. The droop curve under high temperature is like that of an LED with a larger coefficient A while the droop curve under low temperature is like that of an LED with a smaller coefficient A. In Ref. [73], the author used the experimental curves of an LED sample in Ref. [18]. However, the absolute value of EQE was not given in Ref. [18], so fitting on a normalized *EQE*-*J* curve may leave a concern that the fitting parameters are not fully correct. In addition, it was mentioned in Ref. [73] that temperature-dependent EL can directly detect electron leakage by observing additional emission at shorter wavelength near 400 nm, but only at very low temperatures (T < −100 °C) and not near room temperature. The author believed the leakage occurs only at very low temperature, but this is not true. The emission around 400 nm is from the leakage carrier recombination at p-GaN. Under very low temperature, it can be observed as the non-radiative recombination in p-GaN is suppressed, while under room temperature or higher temperature, the leakage carriers recombine dominantly in non-radiative recombination centers because they are thermal-activated. Thus, the fact that the 400-nm leakage peak cannot be observed under room temperature and high temperature does not mean the carrier leakage can be neglected under these temperatures. Noting the LED sample had a very large series resistance of 115 Ω, the author may overestimate the influence of temperature-dependent acceptor activation and hole injection in his simulation but underestimate the influence of temperature-dependent SRH recombination (coefficient *A*). One can see in Ref. [73] that the IQE is as high as 80% at 150 °C. Even if the CIE is 100%, the RRE is still as high as 80% at 150 °C. The coefficient *A* seems too small in his simulation or he might assume a constant coefficient A the same with that under room temperature.

### 3.2. I-V Curve Measurement

**Figure 1:**
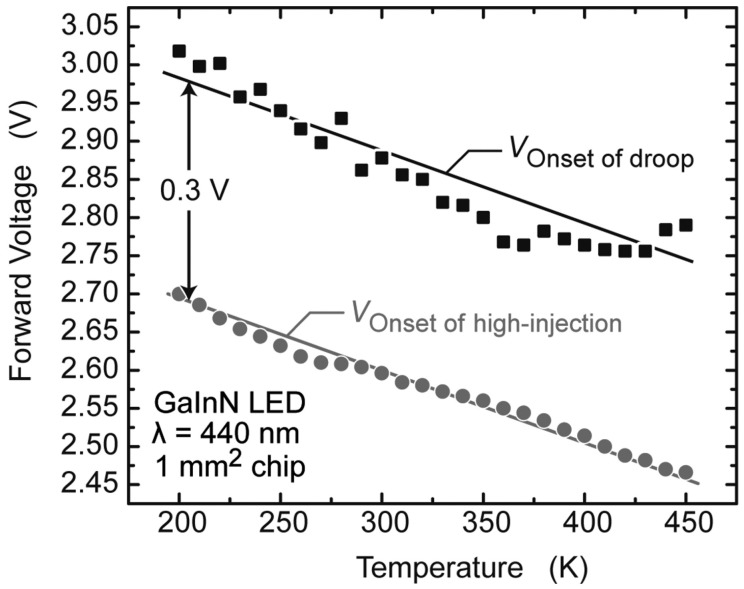
Voltage at the onset of high injection and voltage at the onset of the efficiency droop (i.e., the peak-efficiency point) as a function of temperature. This figure is reprinted from Ref. [51], with the permission of AIP Publishing LLC.

If injection loss is responsible for LED droop, the carrier transportation mechanism should change when droop happens. This change can be reflected in *I*–*V* curve. Meyaard et al. discovered that the efficiency droop is closely related to high injection of carriers [51]. The onset of high injection is defined as the point where the *I*–*V* curve of LED transforms from an exponential slope to a sub-exponential slope. Once the high injection occurs, an electric field is built up in the p-type region, which swaps out electrons, leading to electron leakage and thus, efficiency droop. They examined the *I*–*V* curves of LED under the temperature ranging from 200 K to 450 K, with a step of 10 K. The onsets of high injection of each curve are pointed out according to the definition. These are compared with the onset of efficiency droop where the EQE reaches its peak under corresponding temperatures. It is found that they have a close relationship to each other and the voltage of the onset of efficiency droop is about 0.3 V higher than that of the onset of high injection, in a wide range of temperatures, as shown in Figure 1.

They also explained why the carrier leakage is easy to happen in InGaN/GaN MQW LEDs. If *δ* refers to the ratio of the electron concentration in the barrier to the electron concentration in QW, *δ* = *n*_barrier_*/n*_QW_ ≈ 0.1%. The radiative recombination lifetime in the GaInN QW is in the order of 10 ns. On the other hand, the drift-leakage time (electron sweep-out time) is given by $\tau_{\mathrm{DL}}=\frac{d_{p-\mathrm{GaN}}}{\upsilon_{\mathrm{drift}}}=\frac{d_{p-\mathrm{GaN}}}{\mu_{e}E}$, where *v*_drift_ is the drift velocity, *d*_p-GaN_ is the thickness of the p-type GaN layer (200 nm), *μ*_e_ is the electron mobility in the p-type GaN layer (200 cm^2^/Vs), and *E* is the electric field in the p-type region. The calculated *τ*_DL_ is about 10–500 ps. Since the energy relaxation time in GaN is extremely short, in steady-state, the fraction of electrons leaking from the QW can be expressed by $\frac{n_{\mathrm{barrier}}/\tau_{\mathrm{DL}}}{n_{\mathrm{QW}}/\tau_{\mathrm{rad}}}=\delta \frac{\tau_{\mathrm{DL}}}{\tau_{\mathrm{rad}}}$, which explains why electrons can leak out of the active region, despite the high barriers confining the electrons.

Recently, a new model has been raised by Park et al. [74], wherein the electron leakage depends on the third power of the carrier concentration and an associated third-order drift-leakage coefficient *C_DL_*, which is expressed based on the physical parameters of the pn-junction as

(3) $$C_{DL}=\frac{\delta \mu_{n}}{\mu_{p}p_{p0}}B\;,$$

where *μ*_n_, *μ*_p_, and *p*_p0_ are the electron and hole mobility and equilibrium hole concentration in the p-type layer, respectively. Furthermore, the onset current density of the efficiency droop *J*_onset-of-droop_ can be expressed by the electron leakage model as

(4) $$J_{\mathrm{onset}-\mathrm{of}-\mathrm{droop}}=qd\frac{AB}{C_{\mathrm{DL}}}\;,$$

They compared InGaN-based green and blue LED to investigate the effect of imbalanced carrier concentration and mobility on efficiency droop. Comparison is made between the transport characteristics of blue and green LEDs, which confirms that the asymmetry factor in green LED is higher than that in blue. The *I*–*V* characteristic of both blue LED and green LED are also examined and compared to each other. These comparisons confirmed the electron leakage model, which indicates that the onset of droop current density and the onset of high injection voltage will be lower while the magnitude of efficiency droop will be higher in LED with a greater imbalance in carrier concentration and mobility.

In a recent paper [75], Sorra company reported a best-state-of-the-art LED grown on high quality GaN substrate. It can be seen that in this LED the onset of efficiency droop is around 4–5 A/cm^2^, while the corresponding voltage (*V_f_*) is only around 2.9 V under room temperature. However, the photon energy for this 415-nm LED is nearly 3.0 eV. The authors explained this is caused by the tail of the statistical distribution of injected carriers which enables recombination at energies above *qV_f_* (in other words, carriers absorb energy from phonon scattering before they recombine so that overall energy conservation is satisfied). It seems that droop happens already when voltage is still low. That means droop is more like current density or carrier concentration dependent rather than voltage-dependent. However, this does not conflict with the carrier leakage (injection loss) model. Electrons should obey Fermi distribution in semiconductors, so if high-energy electrons are consumed by some fast process, the carriers in QW have to supplement the high energy electrons. The electric field sweeping is a possible origin to consume the high energy electrons, wherein the electric field is formed by either bias or intrinsic polarization field. In Sorra’s LED sample, even if the bias is lower than built-in voltage, the current has already been unable to neglect (4–5 A/cm^2^). To keep current continuity, there is still a little part of bias added on n-GaN and p-GaN to produce the drift current matching to diffusion current. In addition, Auger-assisted leakage, which is triggered by Auger recombination but is treated as injection loss, is also a possible origin for carrier leakage.

### 3.3. Carrier Injection Efficiency (CIE) Measurement

The experimental determination of CIE is crucial to fully understand recombination mechanisms and causes of efficiency reduction in LEDs. If one can separate RRE and CIE from the EQE, the dominant mechanism of the efficiency reduction can be clarified. In many practical cases, the separation of RRE and CIE is also crucial to clarify the status of the current device. When a specific structure, such as the doping profile, active structures, or electron blocking layers, is modified to optimize the performance, it needs to be answered how much whether the intended modification actually affect the RRE or the CIE or both.

Ahn et al. proposed a method combining EL and photoluminescence (PL) to carry out the CIE measurement [42]. The basic idea is to compare the necessary amount of carriers in optical and electrical excitation to reach the same intensity of light output [76,77]. When the EL is on with a given forward current injection (*J*), an additional laser excitation with 405 nm wavelength illuminates the sample surface. The laser spot is defocused and the spot diameter is twice as large as the sample size in order to illuminate the whole chip surface uniformly. The photon energy of the laser is between the bandgap of the InGaN well and that of the GaN barriers, and consequently all the excitation occurs in the active layers. With this additional laser illumination, the emission intensity increases, and this change in the emission intensity, ΔL_ph_(*J*), is measured.

Let us assume that we know the total number of photogenerated carriers per unit time by the additional laser illumination, Δ*I*_ph-tot_(*J*)/*q*, that actually participate in the recombination process at a given *J*. How to obtain Δ*I*_ph-tot_(*J*)/*q* in detail will be discussed later. Once Δ*I*_ph-tot_(*J*)/*q* is obtained, we then proceed to determine the CIE(*J*) quantitatively. After measuring the EL intensity, EL(*J*), and ΔL_ph_(*J*) at the given *J*, the additional laser is turned off and then the forward current increases until the total electroluminescence (EL(*J* + Δ*J*)) reaches the same intensity as EL(*J*) + ΔL_ph_(*J*), while the current required to reach this intensity is called Δ*I*_EL_(*J*). This measurement process is repeated throughout the range of forward current density. We note here that the required electrical current Δ*I*_EL_(*J*) to reach ΔL_ph_(*J*) is always larger than Δ*I*_ph-tot_(*J*), which means some portion of electrically injected carriers do not participate in the recombination process. The injection efficiency at the given *J* per small carrier density increase can then be determined to be

(5) $$\eta_{inj}=\frac{\Delta I_{\mathrm{ph}-\mathrm{tot}}/q}{\Delta I_{\mathrm{EL}}\left(J\right)/q}\;,$$

Then, the remaining problem would be how to accurately determine Δ*I*_ph-tot_(*J*)/*q*, the total number of photogenerated carriers per unit time by the additional laser illumination that actually participate in the recombination process at a given *J*. The number of carriers generated by the laser in the reverse-biased range was estimated by *I*_ph-sat_/*q*, where *I*_ph-sat_ is the photogenerated current measured at large enough reverse voltage where all the carriers generated escaped from the quantum well. Although *I*_ph-sat_/*q* can give us a good approximation for the number of photogenerated carriers, two correction processes are followed in order to obtain more accurate value of Δ*I*_ph-tot_(*J*)/*q*. One is the possible change in the laser absorption as the current increased in the forward bias region, and the other is not all the photogenerated carriers participate in the recombination process and some portion of the carriers escape from the QWs. Both these calibrations have been reported in Ref. [42].

**Figure 2:**
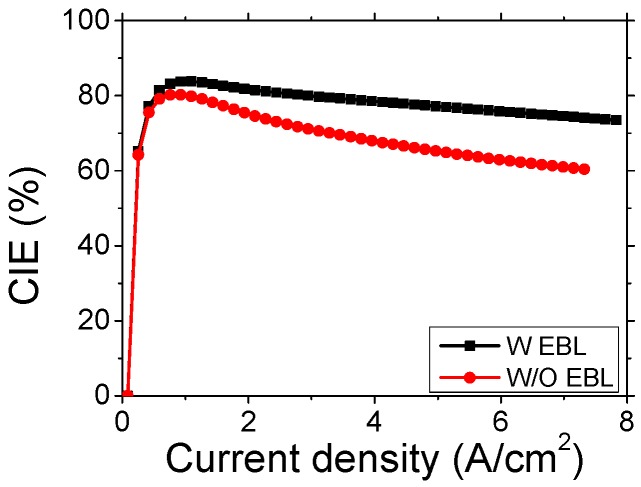
Measured CIE as a function of the injection current density for the samples with-EBL (black) and without-EBL (red) [78]. Courtesy of Prof. Song, J.H.

The effect of electron blocking layer (EBL) in blue-emitting InGaN/GaN LEDs is revealed by using CIE measurement [76]. As shown in Figure 2, the CIEs were 74.5% and 61.0% at 7 A/cm^2^ in samples with and without EBL, respectively. The IE of the sample with EBL is significantly larger than that of without EBL. In addition, they also investigated the LED behaviors under high temperatures (>400 K) [77]. It shows that the carrier leakage is a dominant cause in the efficiency loss at room temperature, while the intrinsic loss such as Auger recombination in the radiative efficiency becomes an important factor in the efficiency loss at high temperature.

## 4. Photoluminescence (PL)

PL from InGaN/GaN MQW can be resonantly excited by a laser with photon energy higher than InGaN bandgap but lower than GaN bandgap, so all the carriers are generated within the QW. Then, the PL intensity can be considered only related to *η*_rad_ of InGaN MQW but with no concern for *η_inj_*. A sublinear increase of PL intensity can be observed as long as the excitation power increases to high enough, which reflects the efficiency droop occurs. By using the *ABC* model to fit the experimental power-dependent PL (PDPL) curves, the role of internal loss can be studied.

Shen et al. used PDPL to obtain the Auger recombination coefficient *C* is in the range of (1.4–2.0) × 10^−30^ cm^6^/s in quasi-bulk In_x_Ga_1−x_N (x ~ 9–15%) layers [12].

However, if considering a carrier delocalization process in InGaN MQW. One can let *C* = 0, and assume coefficients *A* and *B* changing with carrier concentration. If *A* becomes larger when carrier concentration is high, it will also result in efficiency droop. Hader et al. first proposed carrier delocalization as a possible explanation of efficiency droop [29]. Then, Davies et al. discovered, at high excitation power densities, a high energy component in spectra exhibits a more rapidly decay than localization carrier emission, and meanwhile, the RRE also decreases [48].

**Figure 3:**
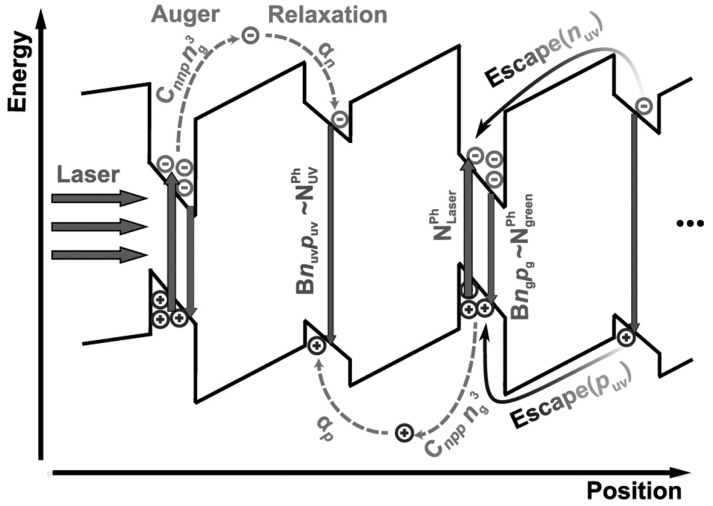
Schematic drawing of the band structure of sample containing alternating green and UV QWs. UV quantum wells are used to capture hot charge carriers generated by Auger processes in the green wells. Luminescence originating from the UV wells therefore visualizes Auger recombination in the green wells. This figure is reprinted from Ref. [52], with the permission of AIP Publishing LLC.

Although either Auger recombination or carrier localization exhibits the similar droop characteristics in PDPL, there should generate high energy carriers in the former. According to this behavior, Binder et al. designed an experiment to directly observe the hot carriers generated by Auger recombination [52]. As shown in Figure 3, they excited a tailored MQW sample containing alternating green (520 nm) and ultraviolet (UV, 400 nm) QWs by using a 450-nm blue laser. Under 12 K, a UV luminescence from the UV QWs could be observed along with the significant droop of emission from the green QWs, indicating that Auger generated hot electrons and holes in green QWs are injected into the UV QWs.

PDPL is a direct evidence that droop can be originated from the internal loss. However, it only validates the internal loss indeed exists in InGaN QW when carrier concentration is high enough. It cannot fully verify the droop appearing in LED EL is the internal loss exactly. There are two concerns. First, the energy bands in PL and EL measurement are different, wherein the former is no-bias while the latter is under forward bias. Second, the excitation power in PL cannot corresponds to injection current in EL directly. Both of them need to transform to carrier concentration according to carrier lifetime. From publications, we can know that the droop in PDPL occurs when excitation power density is higher than 100 MW/cm^2^ [48], but the onset point of EL droop is usually below 10 A/cm^2^ [6]. Thus, there exists a possibility that the onset of droop in EL is before the onset of droop in PL if we compare their carrier concentration.

## 5. Auger Electrons Measurement

Auger recombination is a normal process occurring in semiconductors, especially when the carrier concentration is high. Although there have been many publications believing Auger recombination is a main factor of LED efficiency droop, this microcosmic process was hard to observe, until Iveland et al. achieved a breakthrough recently [54,57]. They designed a negative electron affinity surface by mesh Cesium coating on p-GaN, where the minimum of the conduction band in p-GaN lies above the vacuum level at the surface. Then hot electrons transporting to p-side surface can fly out to vacuum and enter a Faraday cup for energy distribution spectrum analysis. Under high injection, a high energy peak appears, which is believed from L-valley of GaN. Such high energy electrons can be only produced by Auger recombination, after they disproved the possibility that the L and Γ-valley peaks could be generated by photon absorption of LED light and invalidated the possibility that the L-valley peak could be generated by electron heating in the surface band-bending region. This is direct evidence that Auger recombination indeed happens inside the LED. Moreover, it was also found that the onset of droop is synchronous with the appearance of Auger electron peak, though the measured electron emission current is extremely small. Subsequently, Monte Carlo simulations of hot-electron transport carried out by Sadi et al. supported that the experimentally observed hot electrons are created by Auger recombination in QWs [79].

The experimental results seem to answer the long-standing debate perfectly. However, as this experiment takes time and costly efforts, the published data are very limited, which still leaves some unanswered questions. Firstly, the concurrent appearance of Auger electron and the onset of droop should be confirmed in more samples, even including AlGaInP LEDs. The Auger recombination could always occur as an intrinsic phenomenon, even if carrier concentration is low. If the noise of measurement system is suppressed low enough, the Auger electron peak should be observed before the onset of droop. Secondly, there still lacks the sufficiently quantitative analysis on the relation between the measured electron emission current and the droop current component. Thus, we cannot obtain an Auger recombination coefficient from this experiment. Thirdly, according to Ref. [54] and Ref. [57], it was found that the inexistence of a p-AlGaN EBL will lead to the obviously stronger intensity of the high energy electron peak. However, the Auger electrons have high-enough energy, so that the influence of p-AlGaN EBL on their transport should be ignored. Last, the energy band structures of GaN and GaN-based LEDs should be clarified further. In this experiment, the L-valley is found only 0.9-eV higher than Γ-valley, not the conventional 2-eV. However, theoretical simulation of GaN-based avalanche photodiodes using a 2-eV gap agrees well with the experimental result [80]. Thus, the multiple electron peaks appearing in this experiment need further identified because electrons experience many complex hetero-interfaces during their transport in LEDs. Bertazzi et al. also suggested that Auger-excited electrons cannot be unambiguously detected in the LED structures used in Ref. [54] by using a full-band Monte Carlo model based on first-principles electronic structure and lattice dynamics calculations [81]. The measured energy distribution curves are probably uncorrelated with the carrier distribution in the active region, and additional experimental and simulative studies are necessary to unravel the complex physics of GaN cesiated surfaces.

## 6. Leakage Electron Measurement

Profiling temperature or voltage cross the active region of an LED is a direct observation method to understand what happens inside the LED when injection current increases. However, it is extremely challengeable to realize the accurate measurement with high spatial resolution of ~10 nm scale. The scanning thermal microscopy (SThM), which has nano-scale spatial resolution, is most suitable for profiling the temperature the LED’s layers. Jung et al. profiled the temperature distribution on the cross section of the active layer of an LED with a null-point (NP) SThM [60]. The NP SThM can profile temperature quantitatively, which overcomes problems of conventional SThM and can be very sensitive. The principal equation for NP SThM operation is

(6) $$T_{s}\left(x\right)=T_{c1}\left(x\right)-\left(\frac{T_{c2}\left(x\right)-T_{c1}\left(x\right)}{T_{j2}\left(x\right)-T_{j1}\left(x\right)}\right)T_{j1}\left(x\right)\;,$$

which is derived from

(7) $$Q_{\mathrm{ts}}=C\left(T_{\mathrm{nc}}\left(x\right)-T_{c}\left(x\right)\right)\;,$$

(8) $$Q_{\mathrm{ts}}=G_{\mathrm{ts}}\left(T_{c}\left(x\right)-T_{s}\left(x\right)\right)\;,$$

where *T*_s_, *T*_c_, *T*_nc_, *T*_j_, *Q*_ts_, *C* and *G*_ts_ are the temperatures of the sample surface, temperature measured by the SThM probe in contact mode, temperature measured by the SThM probe in non-thermal contact mode, the difference between *T*_c_ and *T*_nc_, heat flux through the tip-sample thermal contact, thermal conductance from the tip of the SThM probe to the surroundings through the probe, and thermal conductance of the tip–sample contact.

It is found that the temperature peak moves towards the p-GaN side as the current density increases, which means more heat is generated in p-GaN. Thus, they believe that at higher current densities, current leakage becomes the dominant cause of the droop.

**Figure 4:**
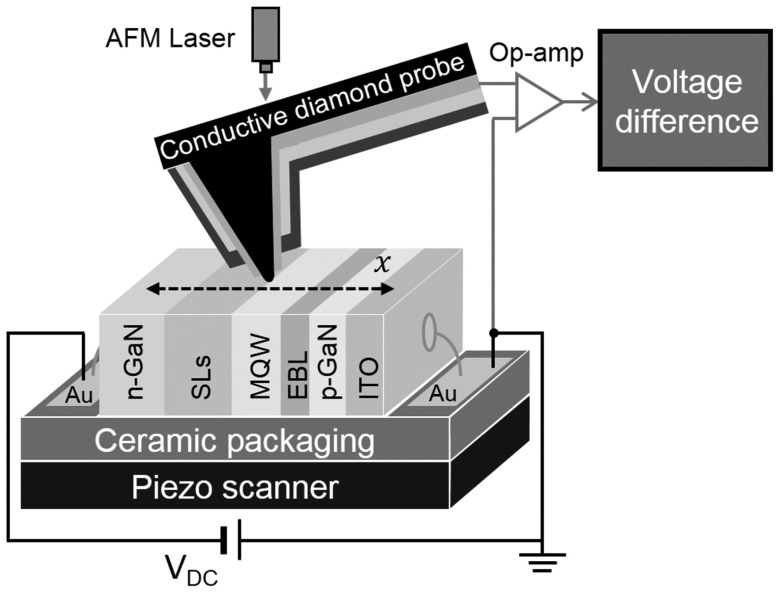
Schematic of the experimental setup for measuring the voltage profile on the cross section of an operating LED. Au electrode of p-GaN side is grounded, the measured voltage drops from 0 V to negative value, approaching n-GaN. This figure is reprinted from Ref. [63], with the permission of AIP Publishing LLC.

The same group also investigated the voltage profiles on the cross section of an operating LED by using the similar setup [63], as shown in Figure 4. As for the voltage, the equation of the total voltage drop of the LED is $V_{F}=V_{d}+R_{S}I_{F}$, where *V*_F_ is the total voltage drop of the LED, *V*_d_ is the bias voltage of its epi-structure, *R*_s_ is the series resistance, and *I_F_* is the injection current. *R*_s_ can be estimated from the slope of the linear regime in *I*–*V* curve under high current density. Under the condition that an external bias is provided, the gradients of the Fermi levels of the electrons and holes occur, mostly in the space–charge region. By analyzing the voltage in different epi-layers of LED under different current density, the movement of carriers can be traced and therefore the status of LED can be find out to examine the causes of efficiency droop.

The voltage profiling study obtains the similar conclusion with temperature profiling that when the current density is low, a steep potential gradient occurs in MQW which indicates that most of the injected electrons are depleted in MQW through radiative recombination. However, when the current density increases, the steep region of potential gradient moves to the EBL and p-GaN, which means the leakage current is the major cause of the efficiency droop at a relatively high current density.

## 7. Carrier Lifetime Measurement

Carrier recombination dynamics can be phenomenologically described by using carrier lifetime *τ*. According to ABC model, the carrier recombination rate can be expressed as

(9) $$\frac{1}{\tau }=A+2Bn+3Cn^{2}\;,$$

Combining Equations (1) and (9), we can see that Auger recombination will enhance the recombination rate that leads to the decline in carrier lifetime, whereas carrier leakage only reduces carrier concentration in the active area and has no effect on carrier lifetime. Additionally, the carrier lifetime also builds a connection between current density *J* and actual carrier concentration *n* in QW as Equation (2) describes. Thus, carrier lifetime acts as a useful tool for droop analysis, as it provides additional effective information besides the efficiency.

Based on differential phase method [82], differential carrier lifetime analysis is an effective means to investigate the recombination characteristics [28,83]. By imposing a small alternating current (AC) component on a direct current (DC) to drive the LED, the carrier lifetime τ is obtained by analyzing the phase delay between the injected AC signal and the emitted light signal. Riuttanen et al. compared *τ* measured by impendence and differential analysis [83]. The longest carrier lifetime was found to be in μs regime, which is considerably larger than commonly reported results [28,84,85]. David et al. utilized the measured *τ* to calculate carrier concentration *n* and subsequently the *A*, *B*, *C* coefficients in *ABC* model [28]. However, the assumption of unit injection efficiency at all injection levels seems inappropriate, and the fitting coefficients *A*, *B*, and *C* are not accurate enough either. Recently, we optimized the measurement system to eliminate the parasitic *RC* constant and obtained the current density-dependent carrier lifetimes in commercial blue and green LEDs [62]. Then the experimental *η*_int_-*J* and *τ*-*J* curves are fitted simultaneously to separate the influence of Auger recombination from that of carrier leakage, which can also eliminate the uncertain in fitting *η*_int_-*J* single curve. We found that the carrier lifetime exhibits a saturated trend in its decline when current density is high, rather than an accelerated decay.

Furthermore, for the state-of-the-art LEDs, the efficiency droop strongly depends on the emission wavelength [6]. Commonly, blue and green LEDs achieve their peak efficiency below 5 A/cm^2^, but the efficiency suffers almost half degradation under 100 A/cm^2^. Green LEDs usually exhibit much more serious droop effect than blue ones. On the other hand, InGaN-based near-UV (365–400 nm) LEDs show a slower efficiency increase as current density raises and reach the maximum under around 10–20 A/cm^2^. Moreover, their droop degree is much weaker, keeping around 60–70% of the peak value under 100 A/cm^2^. This wavelength-dependent (or indium-composition-dependent) phenomenon is so attractive and beneficial to clarify the origins of efficiency droop thoroughly.

We further measure the differential carrier lifetimes of commercialized near-UV LEDs and compare with those of blue and green ones [86]. The commercialized LEDs measured are all grown on standard patterned sapphire substrates, wherein the blue and green ones are the same with those in Ref. [62]. The main structures of LEDs include an n-GaN bulk layer, n-InGaN/GaN superlattices, InGaN/GaN multi-quantum-well (MQW), a p-AlGaN electron blocking layer and a p-GaN contact layer. Their peak wavelengths are around 380 nm, 460 nm and 530 nm, respectively, which are realized by adjusting the indium composition in InGaN QW. The MQW numbers of these samples are around 12–14, while the InGaN QW width keeps around 2.5–3 nm, resulting in the total active region thicknesses of 42 nm, 35 nm, 30 nm in near-UV, blue, and green LEDs, respectively. After growth, face-up chips are fabricated, with the chip size of blue and green LEDs both 300 μm × 300 μm while that of near-UV LED 280 μm × 406 μm. Then, the LED chips are all packaged using resin lenses to enhance light extraction. The injection current-dependent light power, peak wavelength and forward voltage are measured in calibrated integral-spheres for visible LEDs and UV LED, respectively. Thus, EQE (*η*_ex_) of LEDs at different injection current density can be calculated.

As a common sense, LEE (*η*_extr_) changes little with injection current density. Thus, IQE can be represented by *η*_ex_/*η*_extr_, where *η*_extr_ is a constant. In our analysis, *η*_extr_ of near-UV, blue and green LEDs are assumed constant of 50%, 80% and 80%, respectively. The lower *η*_extr_ in the near-UV LED is owing to the absorption of UV light by the thick n-GaN bulk layer and also by ITO transparent electrode and resin lens. If the n-GaN bulk layer is replaced by an n-AlGaN bulk layer or vertical chip fabrication is adopted (light extracted from n-side with wet-etched rough surface), the *η*_extr_ will be increased. Definitely, the IQE may not be exactly accurate, but this does not matter for deriving the conclusion, since the LEE will not change the shape of IQE curves as a constant. Rather than the IQE absolute value, its variation trend is what we really concern in our analysis.

**Figure 5:**
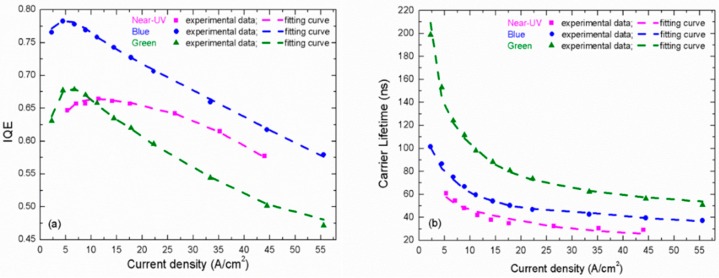
(**a**) IQE; and (**b**) carrier lifetimes depending on injection current density. Dot: experimental data; dash line: fitting curve.

Figure 5a,b shows the experimental IQE (*η*_int_) and carrier lifetimes (*τ*) of near-UV, blue and green LEDs depending on injection current density (*J*), respectively, wherein the IQE *η*_int_ is calculated as *η*_ex_/*η*_extr_. It should be mentioned that the IQE of the near-UV LED seems a bit lower than the state of the art. This is because the IQE of near-UV LED is more sensitive to the dislocation density [87,88]. If instead of GaN buffer layer a sputtered AlN buffer layer is applied to lower the dislocation density [89], the IQE can be further improved to approach or even exceed that of blue one. However, it does not change the variation trend of IQE and the conclusion in following discussion. The *η*_int_ vs. *J* curves shown in Figure 5a are identical to the typical droop behaviors of near-UV, blue and green LEDs, which are usually expressed by normalized plots [6]. In Figure 5b, as the current density increases, the carrier lifetimes’ decreases are rapid first, and then turn slow gradually. One should notice that the carrier lifetime in InGaN LEDs is longer than the conventional III–V semiconductors. As a contrast, a commercial AlGaInP-based LED measured by using the same method has only a carrier lifetime of about 20 ns (not shown). Obviously, the green LED possesses the longest carrier lifetime, while the near-UV one exhibits the shortest. The total carrier lifetime is determined by both radiative recombination lifetime and non-radiative recombination lifetime. The former is influenced by the piezoelectric polarization field in InGaN/GaN MQW [90], which would be stronger when indium composition is higher [91]. The polarization filed tilts the energy band of QW and separates the wave-functions of electron and hole, resulting in wavelength redshift and radiative recombination rate decay [92]. This is so-called the quantum confined Stark effect (QCSE). Due to its highest indium composition, the QCSE in green LED is the most serious among three samples, which is responsible for its longest radiative recombination lifetime [90]. On the other hand, the non-radiative recombination lifetime, which is close related to defects, would be much shorter in near-UV LED than that in blue and green ones. This is because the influence of dislocations on non-radiative recombination becomes more remarkable in near-UV LED due to its lowest carrier localization degree. These reasons lead to the differences of carrier lifetimes in near-UV, blue, and green LEDs shown in Figure 5b. Even if the non-radiative recombination of the near-UV LED can be further suppressed, it will still hold the shortest carrier lifetime due to its weakest QCSE [89].

Similar to that in Ref. [62], a carrier delocalization process is taken into account by introducing the dynamical coefficients *A* and *B* changing with carrier concentration [41]:

(10) $$A=r_{L}\cdot A_{L}+\left(1-r_{L}\right)A_{\mathrm{NL}}\;,$$

(11) $$B=r_{L}\cdot B_{L}+\left(1-r_{L}\right)B_{\mathrm{NL}}\;,$$

(12) $$r_{L}=\frac{1+\exp\left(-\frac{1}{k}\right)}{1+\exp\left(\frac{n-n_{C}}{k\cdot n_{C}}\right)}\;,$$

wherein *A*_L_, *B*_L_ and *A*_NL_, *B*_NL_ represent the SRH and radiative recombination coefficients in localized centers and non-localized centers, respectively. *n*_C_ is the density of localized centers and *k* reflects the state density in these localized centers (Here a larger *k* means a larger size and/or a deeper energy of localized center) [41]. Including this delocalization modification in *ABC* model is crucial to achieve the satisfied fitting results [62].

**Figure 6:**
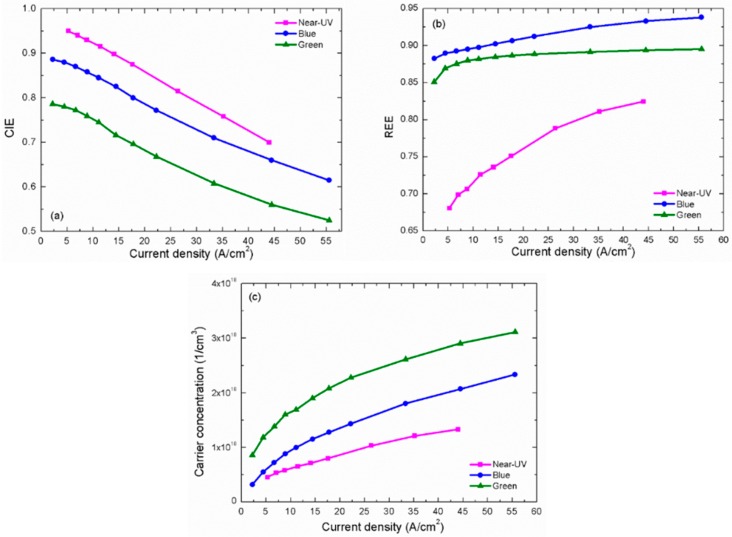
(**a**) CIE; (**b**) RRE; and (**c**) carrier concentration depending on injection current density.

The problem that carrier concentrations in different QWs may be not the same is a limitation. Sometimes in order to avoid this limitation, people choose a SQW LED sample for investigation. However, this will bring another problem that it is not a standard state-of-the-art LED structure. Thus, in our fitting, we have to assume a uniform carrier distribution to calculate the carrier concentration. In a commercial LED, by using the V-pit technique [93], the hole injection uniformity at different QWs can be improved effectively through lateral injection from the sidewalls of V-pit. Thus, the QW number is usually around 10 or even up to 15. Besides, the recent study also indicated that the carrier densities are uniform in different QWs due to the thermally assisted transport [94]. Thus, it is reasonable to assume a uniform carrier distribution and the deviation between this assumption and the actual situation is not as large as expected. According to Equations (1), (2) and (10)–(12), we can obtain the unknown parameters by fitting the *η*_int_ − *J* and *τ* − *J* curves simultaneously. In Ref. [62], it has been shown that if assuming *η*_inj_ = 1 and the efficiency droop is induced only by Auger recombination, no convergent results can be achieved. This is because the experimental 1/*τ* − *n* curves are sublinear, while they should be parabolic if we keep *Cn*^3^, as shown in Equation (9). The above contradiction means the carrier concentration is non-proportional to the current density. In other words, CIE decreases with current density. If we introduce such CIE to calculate the carrier concentration again, it is possible to reshape the 1/*τ* − *n* curve to linear or even parabolic to maintain the reasonability of their relation. If the *Cn*^3^ is really dominant in carrier recombination, it is unlikely that 1/*τ* − *n* curve does not exhibit any parabolic trend. Thus, we choose to believe *Cn*^3^ exists but it can be ignored as the influence of CIE is more important. In our recent paper [95], we used the measured CIE and kept *Cn*^3^ in fitting. The obtained *C* is 1 × 10^−31^ cm^6^ s^−1^, which contributes slightly to efficiency droop. This also indicates more or less that we ignore *C* here is not a big problem. Certainly, the safest treatment is to keep both CIE and *Cn*^3^ in fitting. However, too many fitting parameters will increase the uncertainty of results. Moreover, this is unnecessary because our main purpose is to compare the difference between blue, green and near-UV LEDs and clarify the influence of carrier lifetime. Thus, Auger recombination is ignored for simplicity in following analysis. However, we should emphasize again that it does not mean it is absent and we will still discuss Auger recombination later. Assuming coefficient *C* = 0, the fitting curves coincide exactly with the experimental data, as shown in Figure 5a,b. Meanwhile, *η*_inj_ under different current density (shown in Figure 6a) and the other parameters (shown in Table 1) can be also obtained from the satisfactory fitting results.

In Table 1, we can compare the carrier localization degrees in LEDs with different indium compositions. In previous studies, there is much evidence showing that indium composition fluctuation is the most probable reason for carrier localization and this fluctuation becomes stronger when the indium composition increases [87,92,96]. According to the fitting results, as wavelength of LED changes from near-UV to green, the parameters *n*_C_ and *k* increase from 1.35 × 10^17^ cm^−3^ and 2 to 4.8 × 10^17^ cm^−3^ and 4.4, respectively. As mentioned earlier, *n*_C_ and *k* reflect the carrier localization degree, thus these fitting results are in accord with the well-known common sense, implying the rationality and reliability of the model and the fitting results. Based on the parameters in Table 1, it is easy to calculate the RRE shown in Figure 6b. All the samples exhibit that RRE increases with the current density. This is a merited result when assuming coefficient *C* = 0. The RRE of the present near-UV LED is the lowest among three samples, as it is most sensitive to the dislocation density. Certainly, this is based on the assumption of *C* = 0. The actual RRE will not exactly the same with Figure 6b. It can increase, keep almost unchanged or even decrease with the carrier concentration in our measured range, depending on the value of *C*. However, neither of them is important, because we pay more attention to the trends of CIE in our analysis. This is another limitation of our fitting, but it seems not affect our main conclusion too much.

In Figure 6a, the CIE of all three samples decrease with increased current density, which is also the main cause of efficiency droop. Moreover, the CIE of the near-UV LED is the highest among three samples, while that of the green one is the lowest. This result seems a bit confusing. As the near-UV LED commonly has the shallowest QW, wherein the carrier confinement should be weakest, its carrier leakage should be the most serious among three samples. We will discuss this phenomenon later.

Equation (2) tells us that the carrier concentration *n* in active region of LED is determined not only by current density, but also by CIE and carrier lifetime. Thus, it can calculate *n* through the CIE in Figure 6a and the carrier lifetime *τ* in Figure 5b, and show its dependence on current density *J* in Figure 6c. Although the CIE of the near-UV LED is the highest, its carrier concentration becomes the lowest due to its shortest carrier lifetime. Therefore, Figure 6c exhibits the influence of carrier lifetime on carrier concentration clearly. This means that the actual carrier concentrations in active region of different LEDs possibly vary considerably even though they have the same operating current density and similar active region thicknesses. Both long carrier lifetime and high carrier concentration are not beneficial for good luminescence. The former will increase the possibilities of carrier consumption by non-radiative recombination centers and carrier leakage from QW [51]. The latter not only leads to Auger recombination, but assists carrier leakage as well, because carriers can occupy higher energy levels.

**Figure 7:**
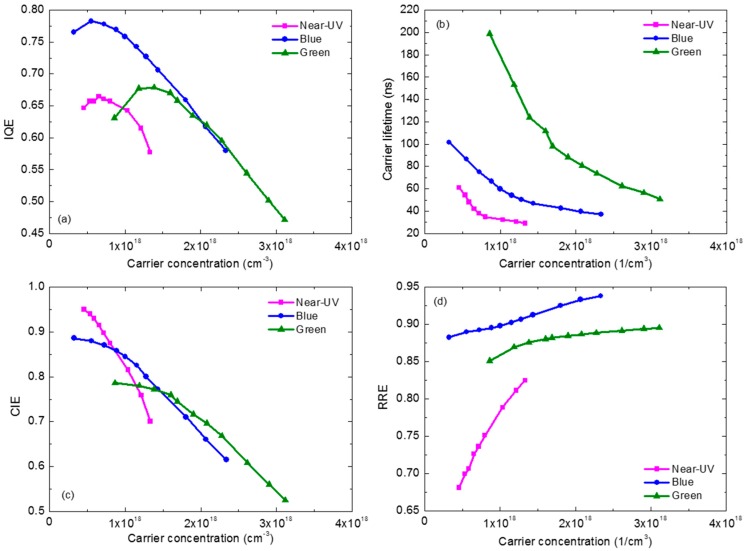
(**a**) IQE; (**b**) carrier lifetime; (**c**) CIE; and (**d**) RRE depending on carrier concentration.

In consideration of carrier concentration’s importance, we plot the IQE, carrier lifetime, CIE and RRE vs. carrier concentration curves, as shown in Figure 7a–d, respectively. The carrier lifetime and the RRE curves follow the similar trends with Figure 5b and Figure 6b, respectively. However, interesting things appear in Figure 7a,c. In Figure 7a, the efficiency droop behaviors in near-UV, blue and green LEDs become totally different. The droop slopes of blue and green LEDs seem almost the same when the IQE is plotted depending on carrier concentration, while in Figure 5a green LED shows stronger efficiency droop effect. Moreover, the near-UV LED has the smallest droop no longer. Its droop degree seems close to or even a bit more serious than that of blue and green ones. Furthermore, in Figure 7c, under the low carrier concentrations, the near-UV LED holds the highest CIE. If we consider the carrier lifetimes under the same low carrier concentrations in Figure 7b, the CIE at this stage is probably determined by carrier lifetime dominantly. In the competition between carrier recombination and carrier leakage, a longer carrier lifetime will lead to the higher possibility of the latter. As carrier concentration increases, however, the CIE of the near-UV LED suffers the rapidest decline. The blue LED is also faced with the similar situation that it shows the earlier CIE droop than the green one. In consequence, under the high carrier concentration, the green LED has the highest CIE instead. Thus, the perplexing CIE picture in Figure 6a becomes clear in Figure 7c. Actually, the shallower the QW is, the more easily it leads to carrier leakage. The depth of QW plays more important role during carrier concentration increasing. The near-UV LED, which has the weakest current-dependent IQE droop, exhibits the most serious carrier concentration-dependent CIE droop, due to its shallowest QW.

Besides the wavelength-dependent efficiency droop behaviors, the carrier lifetime and carrier concentration can also be used to explain another phenomenon that efficiency droop becomes more serious at low temperature [32,37,39,72]. As the non-radiative recombination rate becomes slow, the carrier lifetime will be prolonged at low temperature. This will result in the increase of carrier concentration in active region even under the same injection current. In addition, there have been many publications on improving LED droop performance by a variety of methods. However, we need to re-examine the root cause of these methods: Are they really improving the LED efficiency under the same carrier concentration or just reducing the carrier concentration because of a shortened carrier lifetime? It should be pointed out that decreasing the non-radiative recombination lifetime can also lead to the shorter carrier lifetime, but it will harm the RRE. Sometimes, the droop looks indeed small in poor LED samples [14]. Thus, the normalized efficiency curve is insufficient to validate conclusion and the absolute value of efficiency cannot be ignored when we discuss the droop degree.

Figure 7a,c shows us that the efficiency droop in InGaN-based LED is strongly dependent on carrier concentration. In this sense, current-dependent efficiency droop should actually be carrier concentration-dependent efficiency droop, though the former is more direct-viewing for LED industry. Moreover, the thermal droop is usually defined as efficiency decreasing with the increase of temperature under a fixed current density. However, the precise definition of thermal droop should be temperature-dependent efficiency decay under a constant carrier concentration, because the carrier concentration keeps changing as temperature varies, even at the same current density.

The recent study on IQE measurement by PL methods aiming at the same samples indicated that when carrier concentration is around (0.5–2) × 10^18^ cm^−3^, no droop is observed in PDPL measurement and Auger recombination can be ignored safely [97]. However, as shown in Figure 7a, the EL droop occurs when carrier concentration is only about 0.6 × 10^18^ cm^−3^. This implies the droop is more likely to be triggered by injection loss. However, the above discussion is based on the two assumptions of uniform carrier distribution and ignoring Auger recombination. Their reasonability still requires further confirmation in more samples. Even if this experiment as well as the ones introduced earlier support that the injection loss happens in an operating-LED, it cannot tell what is the exact origin for injection loss. Auger-assisted leakage, which is triggered by Auger recombination but is treated as injection loss, cannot be excluded by these experiments. If it is Auger-assisted leakage, both the carrier recombination rate and the CIE will be influenced. However, according to the carrier lifetime measurement, its influence on recombination rate is unlike the classical *Cn*^3^. If the Auger recombination in GaN-based LED is the defect-assisted Auger [17,35], it might be expressed as *Cn*^2^. In this defect-assisted Auger, an electron transits to a defect energy level in band gap and transfers the energy to another electron in conduction band. The latter electron can transit to a higher energy level in conduction band, and subsequently has great probability to leak from active region. This Auger-assisted leakage will finally result in the poor current injection [23,47]; meanwhile, its influence on carrier lifetime will differ from the classical Auger recombination. These successive defect-assisted Auger recombination and Auger-assisted leakage processes seem quite likely to occur in LEDs according to recent reports on Auger recombination study [54,57]. If it is true, the influence of Auger recombination actually has been included in CIE and *Bn*^2^.

## 8. Solutions to Overcome Efficiency Droop

Increasing the QW width and hence decreasing carrier concentration is a simple way to improve the droop [13,15,16], but it will sometimes bring about the material quality decay due to the accumulated strain. Especially in c-plane LEDs, increasing the QW width will simultaneously strengthen the QCSE, and hence raise the carrier radiative recombination lifetime. From the discussion above, it is found that the intrinsic long carrier radiative recombination lifetime in c-plane InGaN MQW is a fundamental limitation for efficiency droop. Therefore, shortening the carrier radiative recombination lifetime is a more thorough way to overcome the current-dependent efficiency droop. That is why LEDs with low indium composition exhibit weaker efficiency droop effect [75,98]. The possible approaches to overcome efficiency droop include:

(1) Suppressing QCSE in c-plane

For blue or green LED, wherein the intrinsic polarization field is stronger, energy band engineering techniques are desired to shorten the radiative recombination lifetime, for instance, using the staggered QW [99] or thin barrier coupled QWs [20]. Replacing QW by quantum dots (QDs) is also verified to be effective [100,101,102], since the QD relaxes the strain partially and enhance the wave function overlap of electron and hole.

(2) Replacing c-plane by nonpolar/semipolar planes

The nonpolar and semipolar LEDs developed in recent years provide a fundamental solution for QCSE. That is why they always exhibit the excellent efficiency droop-less performance [103,104,105,106,107,108,109], but it still needs more time to reduce their cost.

(3) Introducing additional carrier channel

Additionally, introducing surface plasmons into LED is another possible way to shorten the carrier lifetime [21,30,50], because they provide an extra channel for carrier recombination. However, to prove their practicability in LED industry, it still needs more efforts to eliminate their consequent negative influences on LED’s absolute efficiency.

(4) Changing spontaneous emission to stimulated emission

By introducing a stimulated radiative recombination process, it is believed that laser diode is more attractive than LED in high power lighting [55].

## 9. Conclusions

The experimental measurements for understanding efficiency droop are reviewed. According to the present status, the debate on efficiency droop is not over, though great progresses have been achieved in recent years. The unanswered questions include:

(1) Is the efficiency droop in InGaN-based LEDs more special compared with that in AlGaInP-based red LEDs? Now, carrier lifetime can be used to explain why blue LEDs exhibit more serious droop than red ones. Thus, it is not necessary to be tangled in an abnormal phenomenon to understand why a wide gap semiconductor has a larger Auger coefficient than a narrow gap semiconductor. In this sense, the efficiency droop in InGaN and AlGaInP may possess the same origins.
(2) If Auger recombination is really a dominant factor for LED droop, what is the carrier dynamics model? Obviously, the conventional *Cn*^3^ term goes against the experimental results of carrier lifetime.
(3) If injection loss is really a dominant factor for LED droop, what is the mechanics of carrier leakage? Why do almost all of the optimizing on EBL in LED structure still seem to not solve the problem thoroughly? Is it the Auger-assisted leakage?
(4) How can the IQE, RRE and CIE of a LED be evaluated systematically and accurately without any assumption?

In addition, we also propose some suggestions for future droop study:

(1) It should choose a state-of-the-art LED sample to do measurement.
(2) It should measure an operating LED. PL measurement is not recommended because the droop is EL droop.
(3) It should show absolute efficiency when comparing the droop behaviors. The relative value makes little sense.
(4) It should not assume a 100% current injection efficiency when using *ABC* model.
(5) Apparently, LED droop is current-dependent droop, but physically it is carrier concentration-dependent droop.

## Figures and Tables

**Table 1:** Fitting parameters of near-UV, blue, and green LEDs.

LEDs	*n*_C_ (cm^−3^)	*k*	*A*_L_ (s^−1^)	*A*_NL_ (s^−1^)	*B*_L_ (cm^3^ s^−^^1^)	*B*_NL_ (cm^3^ s^−^^1^)
Near-UV	1.35 × 10^17^	2	1.25 × 10^6^	1.9 × 10^6^	18.5 × 10^−12^	13 × 10^−12^
Blue ^1^	1.5 × 10^17^	2.8	0.25 × 10^6^	1 × 10^6^	14.8 × 10^−12^	6.4 × 10^−12^
Green ^2^	4.8 × 10^17^	4.4	0.17 × 10^6^	1.5 × 10^6^	3 × 10^−12^	2.6 × 10^−12^

^1,2^ Data of blue and green LEDs are from Ref. [62].
